# Grain-Size-Dependent Hydrogen Evolution and Oxygen Evolution Reaction Behavior of a Non-Equiatomic Fe_41_Mn_25_Ni_24_Co_8_Cr_2_ High-Entropy Alloy

**DOI:** 10.3390/ma19091899

**Published:** 2026-05-05

**Authors:** Hee-Tae Jeong, Woo Jin Kim

**Affiliations:** Department of Materials Science and Engineering, Hongik University, 94, Wausan-ro, Mapo-gu, Seoul 04066, Republic of Korea; htjeong1051@g.hongik.ac.kr

**Keywords:** high-entropy alloy, hydrogen evolution reaction, oxygen evolution reaction, grain size, Tafel slope, alkaline electrolysis

## Abstract

**Highlights:**

- Grain refinement was associated with improved apparent OER response.
- HER exhibited a descriptor-dependent correlation with grain size.
- Local Tafel slope most clearly reflected HER grain-size dependence.
- OER showed stronger grain-size dependence than HER in 1 M KOH.
- Microstructural control tuned apparent HER and OER response in bulk HEA.

**Abstract:**

The grain-size dependence of hydrogen evolution reaction (HER) and oxygen evolution reaction (OER) behavior was systematically investigated in a non-equiatomic Fe_41_Mn_25_Ni_24_Co_8_Cr_2_ high-entropy alloy. Six fully recrystallized specimens spanning grain sizes from 5.1 to 197 μm, produced by high-ratio differential speed rolling (HRDSR) and controlled annealing, were tested in 1 M KOH. Differential local Tafel-slope analysis revealed distinct and asymmetric grain-size-dependent behavior for the two half-reactions. For HER, the local Tafel slope at −3 mA cm^−2^ showed the clearest correlation with log(*d*/μm) among the HER descriptors examined in the present dataset (*R*^2^ = 0.682), indicating that grain-size effects were most clearly expressed in the near-onset to intermediate current-density regime. For OER, finer-grained specimens consistently exhibited more favorable apparent performance: the overpotential at 10 mA cm^−2^ increased with log(*d*/μm) (*R*^2^ = 0.715; slope = 1.09 × 10^−2^ V dec^−1^), whereas the current density at an overpotential of 0.33 V decreased with grain size (*j*_0.33_; *R*^2^ = 0.787). Overall, OER showed stronger and more consistent grain-size dependence than HER. These results identify grain size as a useful empirical microstructural descriptor of apparent electrocatalytic response in this composition-fixed bulk HEA system and show that microstructural control provides a practical route for tuning alkaline HER and OER behavior.

## 1. Introduction

Electrochemical water splitting—H_2_O → H_2_ + ½O_2_—is one of the most attractive routes for producing green hydrogen, because it can be powered by intermittent renewable electricity and generates no carbon emissions at the point of use [1]. The overall cell reaction is thermodynamically limited by a standard equilibrium potential of 1.23 V, yet in practice substantially higher voltages are required due to the sluggish kinetics of both half-reactions [2,3]: the cathodic hydrogen evolution reaction (HER) and the anodic oxygen evolution reaction (OER). Minimizing these kinetic overpotentials demands electrocatalysts with high intrinsic activity, large accessible surface areas, and robust long-term stability in the chosen electrolyte.

Platinum group metals—Pt for HER and Ir/Ru oxides for OER—remain state-of-the-art but are prohibitively scarce and expensive for gigawatt-scale electrolysis [4,5,6]. This has prompted intense research into earth-abundant alternatives, ranging from transition-metal phosphides and sulfides [7] to layered double hydroxides [8] and, most recently, high-entropy alloys (HEAs) [9,10]. HEAs are defined by their near-equimolar (or deliberately non-equimolar) incorporation of five or more principal elements in a single solid-solution phase [11]. The resulting multi-element active-site landscape provides a continuous distribution of adsorption energies that can sample optimal values for different reaction intermediates simultaneously, circumventing the linear scaling relations that constrain conventional bimetallic and ternary catalysts [12]. Beyond composition, however, the microstructural dimension of HEA electrocatalysis—specifically the role of grain boundaries (GBs)—has received comparatively little systematic attention.

GBs are planar defects characterized by excess free volume, disrupted local chemical order, and strained lattice [13], all of which are expected to modify the surface electronic structure and local catalytic activity [14]. Severe plastic deformation (SPD) by high-ratio differential speed rolling (HRDSR) followed by controlled annealing provides a uniquely clean pathway to vary grain size over nearly two orders of magnitude while keeping the bulk composition constant [15], thus isolating the microstructural contribution to electrochemical performance. The non-equiatomic Fe_41_Mn_25_Ni_24_Co_8_Cr_2_ alloy studied here is derived from the Cantor HEA family (CoCrFeMnNi) and crystallizes as a single face-centered cubic (FCC) phase [15]. Its non-equiatomic composition was originally designed to balance mechanical performance and corrosion resistance, and the alloy has been extensively characterized microstructurally in prior work [15,16], providing a well-defined materials platform on which grain-size-dependent electrocatalytic behavior can be examined.

HEAs have emerged as promising electrode materials for water electrolysis because their compositional complexity enables broad tunability of catalytic activity and stability [17,18,19,20,21,22,23,24,25,26,27,28,29,30,31,32]. A recent study by Zhang et al. [17] demonstrated a logarithmic dependence of OER performance on grain size in an equiatomic FeCoCrNi HEA, establishing GB density as a viable microstructural descriptor for anodic activity. That work reported an OER regression slope between overpotential and logarithmic grain size of approximately 36–41 mV dec^−1^, and identified a “high-entropy effect” whereby higher-order alloys exhibit weaker grain-size dependence. However, three important questions remain unaddressed. First, it is unclear whether analogous grain-size effects operate for the cathodic HER, and whether the two half-reactions respond to grain refinement in a quantitatively comparable manner within the same alloy system; simultaneous examination of both half-reactions under identical microstructural and electrochemical conditions has not been reported for any HEA system. Second, the extension of this framework to a non-equiatomic five-component composition—specifically one incorporating Mn, which is known to influence surface chemistry and corrosion behavior [16] in Cantor-derived alloys—remains unexplored. Third, no systematic comparison of the electrochemical descriptors most sensitive to grain refinement—overpotential, local Tafel slope, and current density at fixed overpotential—has been carried out across both half-reactions, leaving the choice of microstructural optimization target undefined for bifunctional HEA electrode design.

The present study addresses these gaps by providing a systematic, composition-fixed examination of grain-size-associated HER and OER responses within a single non-equiatomic HEA system. Six fully recrystallized Fe_41_Mn_25_Ni_24_Co_8_Cr_2_ specimens spanning grain sizes from 5.1 to 197 μm, produced by HRDSR and controlled annealing, are tested under identical conditions in 1 M KOH, enabling a direct, composition-neutral comparison of how grain refinement is associated with the apparent responses of the two half-reactions. Specifically, this work addresses two questions: (i) How is grain size correlated with the apparent HER and OER response, and do the two half-reactions exhibit comparable grain-size sensitivity within the same alloy? (ii) Which electrochemical descriptor—overpotential, local Tafel slope, or current density at fixed overpotential—captures the grain-size-associated trend most consistently for each half-reaction?

## 2. Theoretical Background and Analytical Framework

### 2.1. Hydrogen Evolution Reaction (HER) in Alkaline Media

In an alkaline electrolyte, HER proceeds through two possible pathways, both initiated by the Volmer step [33]:

(1) $$Volmer:M+H_{2}O+e^{-}\to M-H^{*}+{OH}^{-}$$

(2) $$Heyrovsky:M-H^{*}+H_{2}O+e^{-}\to H_{2}+{OH}^{-}+M$$

(3) $$Tafel:2M-H^{*}\to H_{2}+2M$$

where M denotes an active surface site, and H* denotes adsorbed hydrogen on an active surface-site. Depending on the catalyst surface and reaction conditions, HER may proceed through either the Volmer–Heyrovsky route or the Volmer–Tafel route [34]. The rate-determining step is often discussed in relation to the Tafel slope (*b*). In alkaline media, canonical Tafel-slope values of approximately 120, 40, and 30 mV dec^−1^ are frequently taken as reference points for Volmer-, Heyrovsky-, and Tafel-limited behavior, respectively [35,36]. However, these values arise from idealized kinetic models and can vary with catalyst composition, surface state, coverage, charge-transfer coefficient, and other non-ideal effects. They should therefore be interpreted as mechanistic reference points rather than strict identifiers, particularly for multicomponent or structurally heterogeneous electrodes.

### 2.2. Oxygen Evolution Reaction (OER) in Alkaline Media

The widely accepted adsorbate evolution mechanism (AEM) for alkaline OER consists of four sequential proton-coupled electron-transfer steps [33]:

(4) $$M+{OH}^{-}\to M-OH+e^{-}$$

(5) $$M-OH+{OH}^{-}\to M-O+H_{2}O+e^{-}$$

(6) $$M-O+{OH}^{-}\to M-OOH+e^{-}$$

(7) $$M-OOH+{OH}^{-}\to M+O_{2}+H_{2}O+e^{-}$$

The theoretical overpotential is governed by the largest free-energy change among these elementary steps. For an ideal catalyst, all four steps would be equally favorable, giving a thermodynamic limiting overpotential of 0.37 V [37]. In some cases, especially on oxidized or reconstructed surfaces, the lattice-oxygen mechanism (LOM), in which lattice oxygen participates directly in O–O bond formation, may also contribute [38]. In the present work, however, OER is interpreted primarily within the AEM framework, and any contribution from LOM is treated only as a plausible mechanistic possibility rather than as a directly verified pathway.

### 2.3. Butler–Volmer Equation and Tafel Analysis

For a single electrochemical step, the current density–overpotential relationship is described by the Butler–Volmer equation [39]:

(8) $$j=j_{0}\left[exp\left(\frac{\alpha_{a}F\eta }{RT}\right)-exp\left(-\frac{\alpha_{c}F\eta }{RT}\right)\right]$$

where *j*_0_ is the exchange current density, *α*_a_ and *α*_c_ are the anodic and cathodic charge-transfer coefficients, *F* is Faraday’s constant (96,485 C mol^−1^), R is the gas constant (8.314 J mol^−1^ K^−1^), and *T* is the absolute temperature. For a simple one-step reaction, *α*_a_ + *α*_c_ ≈ 1, although this relation does not necessarily hold for complex multistep reactions.

At sufficiently large cathodic overpotential, the anodic exponential term becomes negligible, and Equation (8) reduces to

(9) $$\left|j\right|=j_{0}exp\left(\frac{\alpha_{c}F\left|\eta \right|}{RT}\right)$$

which can be written in logarithmic form as

(10) $$\left|\eta \right|=b_{c}\log\left(\frac{\left|j\right|}{j_{0}}\right)$$

where the Tafel slope is

(11) $$b_{c}=\frac{2.303\;RT}{\alpha_{c}F}$$

In this work, the differential (local) Tafel slope is calculated point-by-point from the smoothed polarization curve as

(12) $$b_{c}\left(j\right)=\frac{d\left|\eta \right|}{d\log\left|j\right|}$$

This local representation is more informative than a single global Tafel slope because it captures possible changes in apparent kinetics with current density or overpotential. Such behavior is particularly relevant for multistep reactions and for electrodes whose surface state evolves during polarization. The same differential local-slope framework is therefore applicable to both cathodic and anodic branches.

### 2.4. Grain-Size Scaling Law

For equiaxed grains of mean size (*d*), the GB area per unit volume scales as *k*/*d*, where *k* is ~3.4 [40] for a space-filling tetrakaidecahedral grain geometry (*k* of 2 for the simpler parallel-plate approximation [41]). If catalytic activity increases with the density of GB-derived surface sites, the relevant performance metric (*Y*) is expected, in a first-order approximation, to increase with decreasing grain size, i.e., *Y* ∝ 1/*d*. Accordingly, an inverse-grain-size dependence provides the basic physical expectation. In practice, however, the experimental data for the annealed specimens were represented more conveniently by a linear regression against log(*d*/μm), giving

(13) Y=B+A logd

where *A* and *B* are the fitted slope and intercept, respectively. Thus, Equation (13) should be interpreted as an empirical descriptor of the observed grain-size dependence rather than as a mechanistic rate law. The coefficient of determination (*R*^2^) quantifies how well the measured data follow this empirical log-linear trend.

## 3. Materials and Methods

### 3.1. Material Preparation and Microstructure

The non-equiatomic Fe_41_Mn_25_Ni_24_Co_8_Cr_2_ HEA was produced by vacuum induction melting, homogenized at 1373 K for 13 h, and subsequently processed by HRDSR at room temperature. Six batches of specimens were then annealed at temperatures between 973 K and 1473 K for 1 h under argon atmosphere. The corresponding mean grain sizes were 5.1, 10.2, 26.8, 48, 119, and 197 μm. These grain-size values, together with the supporting electron backscatter diffraction (EBSD) microstructural data, were obtained by re-analysis of previously reported datasets rather than by new EBSD measurements performed in the present study. Full microstructural details, including EBSD inverse pole figure maps, GB-character, and texture data, are available in companion studies [15,16].

Following annealing, all specimens were mechanically ground using SiC papers up to 2000 grit to remove surface oxide formed during heat treatment and to obtain a consistent surface condition prior to electrochemical testing. The specimens were then ultrasonically cleaned in ethanol and dried in air immediately before electrochemical testing. After the final grinding step, all specimens exhibited a metallic luster with no visible surface oxidation before being placed into the electrochemical cell.

### 3.2. Compositional Context

Although the central finding of this work is the grain-size dependence of HER and OER activity, a brief comment on the selected alloy composition is useful. The non-equiatomic Fe_41_Mn_25_Ni_24_Co_8_Cr_2_ alloy was originally designed for mechanical performance [15] and corrosion resistance [16], but its constituent elements are also broadly consistent with compositions often examined in alkaline water-splitting studies [10,42,43,44,45,46]. In particular, Ni- and Fe-containing surfaces are widely associated with OER activity in alkaline media [42,43], while Ni, Fe, and Co have also been discussed as catalytically relevant elements for HER in multicomponent alloy systems [10,44]. Mn and Cr may additionally influence the surface chemistry and electrochemical stability [45,46], although the present study does not attempt to isolate their individual catalytic roles.

In the present work, however, the alloy composition is intentionally held constant for all specimens. Accordingly, the differences in HER and OER responses discussed in Section 4.2 and Section 4.3 are interpreted primarily in terms of microstructural variation, especially grain-size refinement, rather than compositional changes. The role of the present alloy composition should therefore be regarded as a fixed materials platform on which grain-size-dependent electrocatalytic trends can be examined systematically.

### 3.3. Electrochemical Characterization

Electrochemical measurements were conducted in a standard three-electrode cell at room temperature using 1 M KOH electrolyte (pH ≈ 14) and a electrochemical workstation (WonATech Co., Ltd., Seoul, Republic of Korea). The HEA specimen served as the working electrode (geometric area: 1.0 cm^2^); a Pt foil (geometric area: 8.0 cm^2^; WizMAC Co., Ltd., Daejeon, Republic of Korea) and a Hg/HgO reference electrode (1 M KOH filling solution, *E*° = 0.098 V vs. NHE; WizMAC Co., Ltd., Daejeon, Republic of Korea) were used as the counter and reference electrodes, respectively. All potentials are reported on the RHE scale according to

(14) $$E_{RHE}(V)=E_{Hg/HgO}+0.098+0.0591pH$$

Prior to linear sweep voltammetry (LSV), all electrodes were held at 1.6 V vs. RHE for 5 s and then allowed to rest for 5 s before the HER and OER measurements. LSV was performed at a scan rate of 5 mV s^−1^. No iR compensation was applied; the solution resistance was not independently measured, but is expected to have been comparable across all specimens tested under identical electrolyte and cell geometry conditions, so its omission does not affect the relative grain-size trends reported. All measurements represent single runs; replicate measurements were not performed, and therefore no statistical error bars are presented in the polarization curves and Tafel analyses. The differential local Tafel slope was calculated from the Lowess-smoothed (span = 0.10 [47]) polarization curve according to Equation (12).

Because a broad linear Tafel region could not be assumed a priori for the present HEA electrodes, as discussed later in Section 4.2 and Section 4.3, a conventional single-window Tafel fit was not adopted. Instead, the local Tafel slope was used as a condition-specific kinetic descriptor at a given current density. The polarization-derived metrics used in the present analysis were overpotential at fixed current density, local Tafel slope at fixed current density, and current density at fixed overpotential. Because the Tafel slopes were obtained from LSV curves measured at 5 mV s^−1^, they should be interpreted as empirical kinetic descriptors rather than true steady-state mechanistic parameters. Log-linear regressions using Equation (13) were performed only for the six annealed specimens; the as-HRDSRed datum was excluded because its deformation-induced microstructure is not consistent with the GB-based scaling framework used for the annealed series. A Pt foil with a geometric area of 2.0 cm^2^ (WizMAC Co., Ltd., Daejeon, Republic of Korea) was used as the HER benchmark. For the OER benchmark, IrO_x_/carbon-cloth electrodes were fabricated. First, 10 mg of IrO_x_ powder was ultrasonically dispersed for 1 h in a mixed solution of 0.965 mL isopropanol and 0.035 mL of 5 wt.% Nafion solution (Sigma-Aldrich Korea Ltd., Incheon, Republic of Korea). Subsequently, 0.150 mL of the resulting homogeneous ink was drop-cast onto both sides of a carbon cloth substrate with a size of 1.0 cm^2^, followed by mild drying at 333 K for 1 h. The nominal IrO_x_ loading was approximately 1.5 mg cm^−2^ when normalized to the 1.0 cm^2^ geometric area.

## 4. Results

### 4.1. Microstructure of the As-HRDSRed and Annealed Specimens

**Figure 1:**
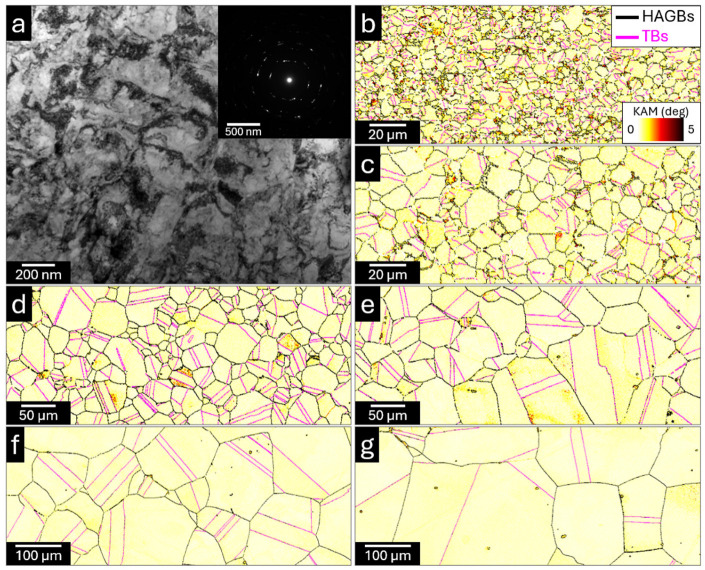
TEM and EBSD microstructural characterization of the as-HRDSRed and annealed specimens. (**a**) TEM micrograph of the as-HRDSRed specimen, with the inset corresponding to the selected-area electron diffraction pattern. (**b**–**g**) EBSD micrographs of specimens after 1 h annealing at (**b**) 973 K, (**c**) 1073 K, (**d**) 1173 K, (**e**) 1273 K, (**f**) 1373 K, and (**g**) 1473 K. In the EBSD maps, black lines denote high-angle grain boundaries (HAGBs) with misorientation angles greater than 15°, whereas pink lines denote twin boundaries (TBs). The microstructural color represents the kernel average misorientation (KAM) angle, according to the color scale shown in (**b**).

**Figure 2:**
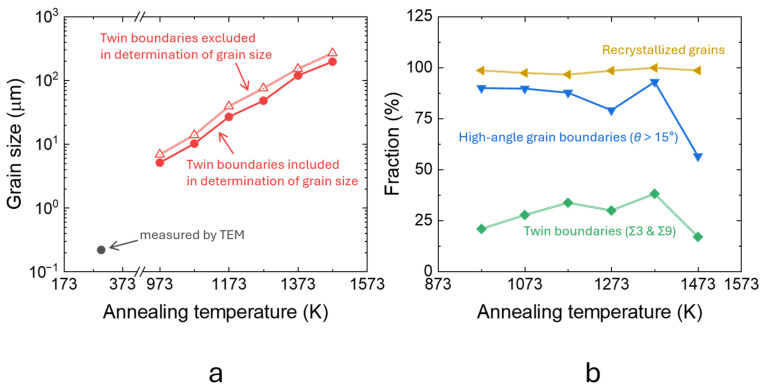
Grain size and boundary characteristics as a function of annealing temperature. (**a**) Grain size as a function of annealing temperature compiled from TEM and EBSD measurements. Grain sizes determined from EBSD are shown with twin boundaries either included or excluded in the grain-size calculation. (**b**) Fractions of high-angle grain boundaries, twin boundaries, and recrystallized grains as a function of annealing temperature.

Figure 1a–g summarize the microstructural evolution of the as-HRDSRed and annealed specimens, while Figure 2a,b presents the corresponding grain size, boundary-character distribution and recrystallized fraction. The as-HRDSRed condition (Figure 1a) exhibits a heavily deformed ultrafine-grained structure with a sub-micrometer intercept grain size of 0.22 μm (Figure 2a), consistent with the ring-like selected-area electron diffraction pattern. Because this deformation-induced state does not represent a well-developed annealed boundary network, EBSD-based boundary fractions are not reported for this condition. After annealing, the microstructure becomes nearly fully recrystallized over the entire temperature range of 973–1473 K, as indicated by the recrystallized fraction remaining close to 100% in Figure 2b.

The EBSD maps in Figure 1b–g show progressive grain coarsening with increasing annealing temperature. High-angle grain boundaries (HAGBs; *θ* ≥ 15° where *θ* is misorientation) constitute the dominant boundary type throughout the annealed series, with fractions ranging from approximately 56% to 93% and reaching a maximum at 1373 K. Twin boundaries (TBs; Σ3 and Σ9) also evolve systematically, increasing from about 21% at 973 K to about 39% at 1373 K, followed by a decrease at 1473 K. This trend is consistent with the development of annealing twins during grain growth in FCC alloys. The apparent decrease in both HAGB and TB fractions at 1473 K should, however, be interpreted with caution, because the very coarse grain size substantially reduces the number of grains captured within the EBSD scan area and therefore increases the statistical uncertainty of the measured boundary fractions.

The KAM angle distributions (Figure 1b–g) further indicate that local misorientation is generally low in the annealed specimens, consistent with the predominance of recrystallized grains. Relatively higher KAM values are localized mainly near some HAGBs and triple-junction regions, whereas most grain interiors remain at low KAM levels. This confirms that the annealed specimens contain little residual intragranular strain compared with the as-HRDSRed state, and that the main microstructural change across the annealed series is not retained deformation but progressive grain growth accompanied by boundary-network evolution. In this regard, grain size remains the primary varying microstructural parameter across the annealed specimens, while the evolution of TBs represents a secondary but systematic change in boundary character. These observations provide the basis for discussing the electrochemical trends in Section 4.2 and Section 4.3 primarily in terms of grain-size-associated microstructural variation, while treating boundary-density effects as a plausible but not uniquely verified contributor.

### 4.2. HER Performance

**Figure 3:**
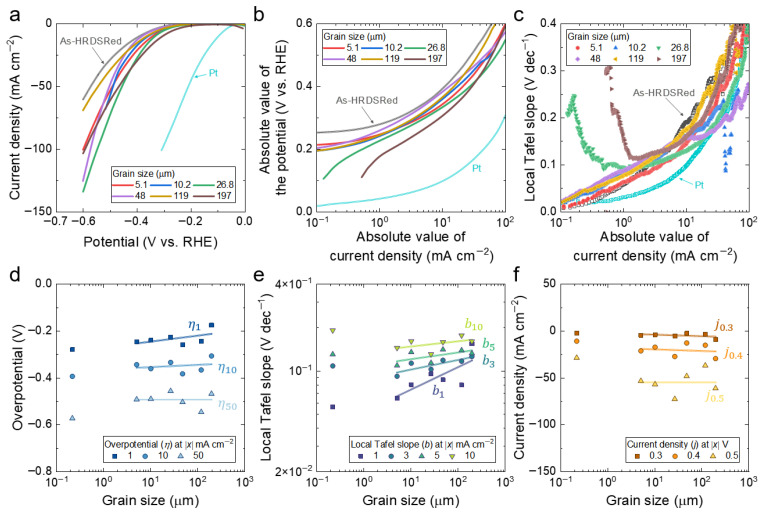
Grain-size-dependent HER polarization behavior of the non-equiatomic HEA in alkaline electrolyte. (**a**) HER polarization curves plotted as current density versus potential (V vs. RHE) for the as-HRDSRed and annealed specimens with different grain sizes, together with the Pt benchmark. (**b**) Absolute value of the potential as a function of the absolute value of current density. (**c**) Local Tafel slope plotted against the absolute value of current density. (**d**) Grain-size dependence of the overpotential at current densities of 1, 10, and 50 mA cm^−2^. (**e**) Grain-size dependence of the local Tafel slope evaluated at current densities of 1, 3, 5, and 10 mA cm^−2^. (**f**) Grain-size dependence of the current density measured at overpotentials of 0.3, 0.4, and 0.5 V.

Figure 3a presents the cathodic polarization curves of the annealed specimens together with the as-HRDSRed reference and a Pt benchmark. The HEA curves exhibit broadly similar cathodic polarization shapes and remain relatively close to one another, whereas Pt is distinctly shifted toward lower overpotential, achieving ∣*η*_10_∣ ≈ 101.3 mV under the same conditions. Within the annealed series, the relative positions of the curves change with current density, indicating that HER performance does not follow a simple monotonic trend with grain size across the full current-density range. The as-HRDSRed specimen lies above the annealed curves over much of the measured potential range, indicating a weaker cathodic response.

The semi-logarithmic representation (Figure 3b) provides a clearer view of the low-to-intermediate overpotential regime. However, none of the HEA specimens, including the as-HRDSRed condition, exhibits a well-defined linear Tafel region extending over at least one decade. This indicates that a single apparent Tafel slope cannot fully represent the overall HER polarization response of the present alloy system. Instead, Figure 3b reveals only a limited quasi-linear segment, from which apparent global Tafel slopes of about 120–140 mV dec^−1^ may be estimated for the annealed specimens. These values fall within the range commonly discussed in the context of alkaline HER kinetics, but given the absence of a well-defined linear Tafel region over at least one decade, no reliable rate-determining step assignment is made for the present multicomponent electrode system. Pt, by contrast, shows substantially lower Tafel slopes, reflecting its much faster HER kinetics.

The differential local Tafel slope (Figure 3c) provides a more detailed description of the apparent kinetic response as a function of current density. In contrast to a single global slope, the local representation captures the continuous evolution of the polarization behavior with increasing cathodic driving force. For the HEA specimens, the local Tafel slope increases gradually with current density and then rises more sharply in the high-current regime. If a single, well-defined Tafel slope governed the reaction over the entire current range, the local Tafel slope would appear as a constant value, i.e., a horizontal line in Figure 3c. The observed variation therefore indicates that the HER response cannot be described by a single kinetic regime but instead reflects a progressive change in the governing processes with increasing overpotential.

The grain-size dependence of the HER descriptors is summarized in Figure 3d–f, where the as-HRDSRed datum is shown for comparison but excluded from the log-linear regressions, which were performed only for the six annealed specimens. The corresponding regression parameters for the annealed series are summarized in Table 1. This distinction is important because the as-HRDSRed condition represents a deformation-induced microstructure rather than the recrystallized grain-size series defined by the annealed specimens. Overall, the annealed series indicates that grain-size sensitivity is expressed most clearly in the overpotential and local Tafel-slope descriptors at low-to-intermediate current density, whereas the current-density descriptors at fixed overpotential show much weaker and less systematic dependence.

The grain-size scaling of overpotential is shown in Figure 3d. In this notation, the overpotential evaluated at an absolute value of current density of *x* mA cm^−2^ is denoted by *η_x_*. For the six annealed specimens, the log-linear fits indicate that the grain-size dependence is strongest at low current density and weakens progressively as the cathodic driving force increases. In particular, *η*_1_ shows the clearest positive regression slope with grain size, whereas the slope becomes much weaker for *η*_10_ and approaches near-flat or reversed behavior at *η*_50_. This trend indicates that the beneficial effect of grain refinement on HER overpotential is expressed most clearly near onset, but becomes increasingly obscured at higher current density. The scatter in *η*_10_ and the more pronounced non-monotonicity in *η*_50_ are consistent with this weakening trend. A plausible contribution is enhanced H_2_ bubble accumulation on fine-grained surfaces, where the higher density of boundary-intersecting surface sites may promote bubble nucleation and local coverage under strong cathodic polarization [48,49]. This would tend to obscure the intrinsic grain-size effect at higher current density and contribute to the weaker or non-monotonic scaling observed for *η*_10_ and *η*_50_. Although the as-HRDSRed datum was excluded from the regression analysis, it consistently exhibits more negative signed overpotential than the annealed specimens at the corresponding current densities. This indicates that the deformation-induced microstructure gives rise to a distinct HER response that is not captured by the grain-size-based scaling relation established for the annealed series.

A clearer grain-size dependence emerges for the local Tafel-slope descriptors in Figure 3e. Here, the local Tafel slope evaluated at an absolute value of a current density of *x* mA cm^−2^ is denoted by *b_x_*. As summarized in Table 1, *b*_3_ gives the highest coefficient of determination, although the numerical difference from adjacent descriptors is not large enough to define a sharply unique optimum. More generally, the intermediate current-density window as a whole shows the most consistent grain-size correlation among the HER metrics examined. The positive regression slope indicates that the local Tafel slope increases systematically with increasing grain size, suggesting that finer-grained annealed specimens exhibit more favorable apparent HER kinetics in this regime. In contrast to the overpotential descriptors, however, the as-HRDSRed datum in Figure 3e deviates from the annealed grain-size trend in the direction of higher local Tafel slope. Its values are therefore generally larger than would be expected from the regression defined by the annealed specimens, indicating that the deformation-induced microstructure does not follow the same grain-size-dependent scaling relation when assessed by the local Tafel-slope descriptors.

By contrast, the current-density descriptors at fixed overpotential (*j*_0.3_, *j*_0.4_, and *j*_0.5_; Figure 3f) show weak or negligible grain-size dependence, with substantial scatter and no consistent monotonic trend. This behavior indicates that, at large cathodic driving force, intrinsic grain-size effects are largely obscured by other contributions, such as interfacial coverage changes, bubble accumulation, and possible transport-related limitations. The as-HRDSRed datum also falls within this scattered distribution and does not exhibit a distinct response in this representation.

Taken together, these results show that the deviation of the as-HRDSRed condition from the annealed grain-size trend is descriptor-dependent: it exhibits lower-than-expected overpotential in Figure 3d, but higher-than-expected local Tafel slope in Figure 3e. This contrast indicates that the deformation-induced microstructure cannot be interpreted simply as an extension of the annealed grain-size series, but instead represents a distinct HER response depending on the descriptor considered. Among the annealed specimens, the low-to-intermediate current local Tafel-slope descriptors show the strongest grain-size dependence, with *b*_3_ giving the highest *R*^2^ value. However, this result should be interpreted cautiously because the representative descriptor was identified post hoc by comparing multiple current-density conditions on the same dataset of only six specimens. The intermediate current-density regime should therefore be regarded as a window in which grain-size effects are collectively more visible, rather than as a uniquely optimal operating point established by independent validation.

### 4.3. OER Performance

**Figure 4:**
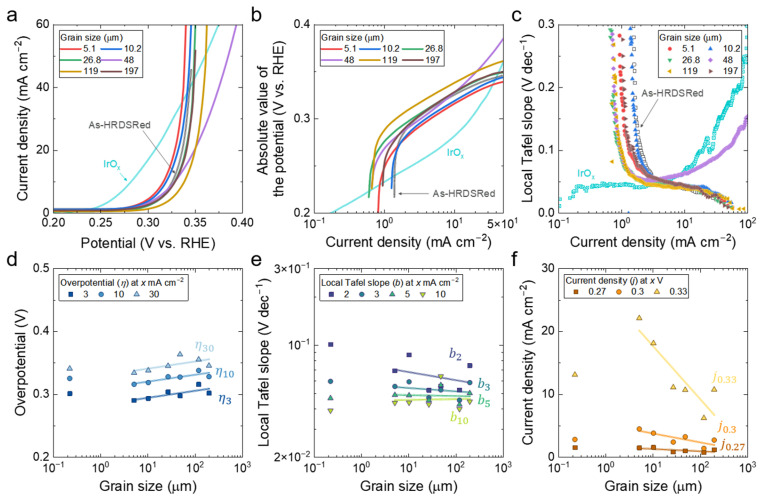
Influence of grain size on the OER electrocatalytic performance of the non-equiatomic HEA in alkaline electrolyte. (**a**) OER polarization curves of the as-HRDSRed and annealed specimens with different grain sizes, presented as current density versus potential (V vs. RHE), with IrO_x_ included as a benchmark catalyst. (**b**) Potential plotted as a function of current density. (**c**) Local Tafel slope as a function of current density. (**d**) Grain-size dependence of the overpotential at current densities of 3, 10, and 30 mA cm^−2^. (**e**) Grain-size dependence of the local Tafel slope evaluated at current densities of 2, 3, 5, and 10 mA cm^−2^. (**f**) Grain-size dependence of the current density measured at overpotentials of 0.27, 0.30, and 0.33 V.

The OER polarization behavior of the HEA specimens is summarized in Figure 4a–c. Figure 4a presents the anodic polarization curves of the annealed specimens together with the as-HRDSRed reference and an IrO_x_ benchmark. The HEA curves exhibit broadly similar anodic polarization shapes and remain relatively close to one another, whereas IrO_x_ is distinctly shifted toward lower overpotential, reflecting its superior OER activity; IrO_x_ achieved *η*_10_ ≈ 282 mV under the same conditions. Within the annealed series, the relative positions of the curves vary systematically with grain size, with finer-grained specimens generally showing higher current density at a given potential.

The semi-logarithmic representation (Figure 4b) provides a clearer view of the low-to-intermediate overpotential regime. As in the HER case, however, none of the HEA specimens, including the as-HRDSRed condition, exhibit a well-defined linear Tafel region extending over at least one decade. Thus, a single apparent Tafel slope cannot adequately represent the full OER polarization response of the present alloy system. Instead, Figure 4b reveals only limited quasi-linear segments, consistent with the heterogeneous nature of the multicomponent surface and the coexistence of multiple reaction pathways with different apparent kinetics.

The differential local Tafel slope (Figure 4c) provides a more detailed description of the apparent anodic kinetic response as a function of current density. In contrast to a single global slope, the local representation captures the continuous evolution of the polarization behavior with increasing anodic driving force. The local Tafel slope varies continuously with current density, indicating that the OER response cannot be described by a single kinetic regime over the full range examined.

The grain-size dependence of the OER descriptors is summarized in Figure 4d–f. Although the as-HRDSRed datum is included in the plots for comparison, the log-linear regressions were performed only for the six annealed specimens. The corresponding log-linear regression parameters for the annealed OER descriptors are summarized in Table 2.

The grain-size scaling of overpotential is shown in Figure 4d. The overpotential evaluated at a current density of *x* mA cm^−2^ is denoted by *η_x_*. For the six annealed specimens, the fitted trends indicate that overpotential decreases systematically with decreasing grain size, meaning that finer-grained specimens exhibit a more favorable apparent OER response. This tendency is observed consistently for *η*_3_, *η*_10_, and *η*_30_, indicating that grain refinement enhances anodic activity in a relatively robust manner across the examined current-density range. Although the as-HRDSRed datum was excluded from the regression analysis, it does not conform to the annealed grain-size trend, again indicating that the deformation-induced microstructure should not be interpreted simply as an extension of the recrystallized annealed series.

The local Tafel-slope descriptors in Figure 4e show a much weaker and less consistent grain-size dependence than the overpotential metrics. As summarized in Table 2, the fitted slopes are small overall and the corresponding correlations are weak, with both low *R*^2^ values and a sign change at higher current density. This indicates that the local Tafel slope is not a robust descriptor of grain-size sensitivity for OER in the present alloy system.

The current-density descriptors at fixed overpotential are shown in Figure 4f. The current density determined at an overpotential of *x* V is denoted by *j_x_*. All three descriptors exhibit strong grain-size dependence in the regression analysis, with *j*_0.33_ giving the highest coefficient of determination among the OER metrics. The remaining descriptors, *j*_0.3_ and *j*_0.27_, also show substantial log-linear correlations, indicating that the grain-size effect on OER is robust across the fixed-overpotential descriptors and becomes most strongly expressed at higher anodic driving force.

Taken together, the results summarized in Table 2 show that OER in the present alloy system exhibits consistently strong grain-size dependence when evaluated using overpotential-based and fixed-overpotential current descriptors, whereas the local Tafel-slope descriptors provide only weak and inconsistent correlations. Thus, unlike HER—where the intermediate-current local Tafel-slope descriptors most clearly resolve grain-size effects—the OER response is more directly and robustly captured by overpotential-based metrics.

## 5. Discussion

### 5.1. Grain Size as an Empirical Microstructural Descriptor of Apparent Electrochemical Response

Within the present annealed series, the most defensible first-order interpretation is empirical rather than mechanistic. All specimens share an identical nominal composition, are nearly fully recrystallized, and were tested under identical electrochemical conditions. Under these constrained conditions, grain size provides the most systematic and experimentally controlled variable across the dataset. Accordingly, the electrochemical trends observed in Section 4.2 and Section 4.3 are most appropriately described as a correlation between grain size and the apparent electrochemical response, rather than as direct evidence of a specific microscopic mechanism.

A GB-density-based interpretation remains physically plausible, because grain refinement increases the interfacial area associated with GBs, which may differ from grain interiors in atomic coordination, defect density, short-range chemical inhomogeneity, and residual strain state [50,51]. However, in the absence of electrochemically active surface area (ECSA) measurements or post-test surface characterization, it is not possible to distinguish whether the observed trends arise primarily from an increase in the number of active sites (geometric effect) or from changes in intrinsic activity per site (electronic or chemical effects). The present results therefore do not uniquely establish GBs as the dominant active sites, but instead identify grain size as a robust empirical descriptor of the apparent activity under the conditions examined.

In this context, all current densities are normalized to the geometric electrode area (1.0 cm^2^), and the reported trends should be interpreted as apparent activity per geometric area. This convention enables consistent comparison across specimens but does not provide direct access to intrinsic activity normalized by the true active surface area. Consequently, the grain-size dependence reported here should be understood as an experimentally observed correlation within a composition-fixed system, rather than as a definitive mechanistic attribution to GB-mediated catalysis.

### 5.2. Distinct Role of the As-HRDSRed State

A particularly informative comparison is provided by the as-HRDSRed specimen. This condition does not simply extend the annealed grain-size series toward the ultrafine limit. Rather, it represents a deformation-induced state characterized by very high defect density and sub-nanometer crystallite scale, and its electrochemical response deviates from the annealed log-linear trends in a descriptor-dependent manner. In the near-onset regime and for some overpotential-based metrics, it tends to outperform the annealed regression, whereas this advantage diminishes at higher current density and, for some descriptors, falls below the extrapolated annealed trend. This behavior indicates that the deformation-induced microstructure should not be interpreted merely as “smaller grain size,” but as a distinct catalytic state in which non-equilibrium defects and interfaces affect the electrochemical response differently from the more regular HAGB network of the annealed series.

This distinction is important for the interpretation of microstructural engineering in bulk HEA electrodes. The annealed series provides a controlled framework in which grain size and GB density can be treated as the dominant varying parameters. The as-HRDSRed specimen, by contrast, highlights that severe deformation introduces a different class of defects and interfaces that may enhance near-onset response but do not necessarily provide the same descriptor-consistent behavior across the broader kinetic window. This is precisely why the as-HRDSRed datum was included in the plots for comparison but excluded from the log-linear regressions.

### 5.3. Descriptor-Dependent Grain-Size Sensitivity in HER and OER

One of the clearest outcomes of the present work is that the grain-size effect is strongly descriptor-dependent, and that this descriptor dependence differs between HER and OER. For HER, grain refinement is expressed most consistently not as a monotonic improvement in high-current overpotential, but as a reduction in the local Tafel slope in the near-onset to intermediate current-density regime. In the annealed series, the local Tafel-slope descriptor (*b*_3_) shows the strongest correlation with grain size within the present dataset, whereas the overpotential descriptors become progressively less reliable as the cathodic driving force increases. This difference may reflect the current-density window in which each descriptor is evaluated. At low-to-intermediate current density, the measured HER response may more directly reflect interfacial reaction kinetics, so that the microstructural effect of grain refinement becomes more clearly expressed. By contrast, at higher cathodic driving force, the measured overpotential may increasingly include additional contributions such as local interfacial coverage [48,49] and transport-related perturbations [49,52]. These effects may obscure the apparent grain-size dependence, making high-current overpotential a less selective descriptor of the microstructural contribution. In this sense, *b*_3_ may represent a kinetic window in which the grain-size effect is more clearly resolved because the response is less strongly influenced by high-current secondary effects.

For OER, the picture is different. The grain-size dependence remains evident across a broader anodic window, and the most robust correlations are obtained not from the local Tafel slope but from overpotential-based descriptors and current density at fixed overpotential. In particular, *η*_10_ and *j*_0.33_ show the strongest correlations with grain size, whereas the local Tafel-slope descriptors are weak and inconsistent. This suggests that, in OER, grain refinement is more clearly expressed in descriptors associated with the magnitude of the anodic response than in the local Tafel-slope descriptors. In other words, HER and OER are both grain-size sensitive, but they express that sensitivity through different descriptor families. This distinction may arise in part because overpotential-based metrics and local Tafel-slope descriptors do not quantify the same aspect of the polarization response. Overpotential at a fixed current density reflects the cumulative voltage cost required to reach a given current density and therefore may incorporate multiple contributions, including interfacial kinetics, surface coverage effects [48,49], and transport-related perturbations [49,52]; although the relative magnitudes of these contributions were not independently quantified. By contrast, the local Tafel slope represents the differential response, that is, the local variation of overpotential with respect to log∣*j*∣, and is therefore more sensitive to the local shape of the polarization curve within a specific current-density window. As a result, the two descriptor families may respond differently to the same underlying microstructural change, especially when nonlinear contributions become significant.

One plausible interpretation is that HER, which involves a single key adsorbed intermediate (H*), may express microstructural effects more directly through kinetic descriptors such as the local Tafel slope. By contrast, OER proceeds through sequential intermediates (OH*, O*, OOH*), and the measured grain-size dependence may therefore become more clearly expressed in descriptors related to the overall anodic response, such as overpotential and current at fixed overpotential. This mechanistic asymmetry is qualitatively consistent with the observed descriptor-dependent grain-size sensitivity, although direct verification would require surface-sensitive characterization of the post-reaction electrode state.

### 5.4. Mechanistic Implications, Limitations, and Practical Significance

The present data establish a consistent empirical relationship between grain size and the apparent electrochemical response of the annealed Fe_41_Mn_25_Ni_24_Co_8_Cr_2_ alloy. Grain refinement is associated with improved apparent performance; however, the manner in which this improvement is expressed depends on both the electrochemical reaction (HER vs. OER) and the descriptor used to quantify performance. These observations should therefore be interpreted within the limitations of the present experimental framework.

First, no post-test surface characterization or ECSA measurement was performed. As a result, the present dataset does not allow separation of geometric effects from intrinsic catalytic effects. The mechanistic interpretations discussed above, such as possible contributions from interfacial coverage effects, bubble accumulation, or transport-related perturbations, are consistent with the literature [48,49,52] but were not directly tested in this study. They should therefore be regarded as physically plausible interpretations rather than experimentally verified mechanisms.

Second, the use of local Tafel slopes as kinetic descriptors requires careful interpretation. Because no extended linear Tafel region is observed in the polarization curves, the extracted local slopes represent condition-dependent differential responses derived from LSV data, rather than true steady-state mechanistic parameters. Accordingly, these values are best treated as empirical kinetic indicators that enable comparison within the present dataset, rather than as definitive indicators of rate-determining steps.

Despite these limitations, the present results retain practical significance. By holding composition constant and varying microstructure through a scalable processing route (HRDSR + annealing), the study demonstrates that grain size can serve as a controllable design parameter for tuning the apparent HER and OER response of a bulk high-entropy alloy electrode. The key implication is not that a specific microscopic mechanism has been uniquely identified, but that microstructural control provides a reproducible and practically accessible pathway for modifying electrochemical performance in compositionally complex alloys.

## 6. Conclusions

The grain-size dependence of the hydrogen evolution reaction (HER) and oxygen evolution reaction (OER) in the non-equiatomic Fe_41_Mn_25_Ni_24_Co_8_Cr_2_ high-entropy alloy was systematically evaluated over a grain-size range of 5.1–197 μm in 1 M KOH. The main conclusions are as follows.

1. OER activity showed a clear correlation with grain size across the annealed Fe_41_Mn_25_Ni_24_Co_8_Cr_2_ series, with finer-grained specimens exhibiting more favorable apparent anodic response at constant composition.
2. HER showed a more descriptor-dependent grain-size response. Grain refinement was associated with improved local Tafel-slope response, but did not produce a monotonic decrease in HER overpotential over the full grain-size range.
3. For HER, the local Tafel-slope at −3 mA cm^−2^ (*b*_3_) showed the strongest correlation with grain size among the evaluated HER descriptors within the present dataset, indicating that grain-size effects were most clearly expressed in the near-onset to intermediate current-density regime. Because this descriptor was selected post hoc from a six-specimen annealed dataset, it should be regarded as a representative empirical indicator rather than a validated universal HER descriptor.
4. For OER, the current density at an overpotential of 0.33 V (*j*_0.33_) showed the highest correlation among the evaluated OER descriptors within the present dataset, while *η*_10_ also exhibited a robust grain-size-associated trend. Overall, OER displayed stronger and more consistent apparent grain-size sensitivity than HER.
5. The as-HRDSRed specimen did not behave simply as an ultrafine extension of the annealed series. In HER, it showed lower-than-expected overpotential for some current-density conditions but higher-than-expected local Tafel-slope values relative to the annealed regression trend, indicating a distinct deformation-induced electrochemical state.
6. Overall, the present results show that grain size serves as a useful empirical microstructural descriptor of apparent alkaline HER and OER response in this bulk HEA system, and that microstructural control via HRDSR and annealing provides a practical strategy for tuning electrocatalytic behavior without changing alloy composition.

## Figures and Tables

**Table 1:** Log-linear regression parameters for HER performance metrics as a function of grain size. The regression was performed using *Y* = *A* log(*d*/μm) + *B* for annealed specimens, excluding the as-HRDSRed specimen. * indicates a representative HER descriptor in the intermediate current-density regime of 2–5 mA cm^−2^.

Parameter	Fixed Condition	Symbol	Slope, *A*	Intercept, *B*	*R* ^2^
Overpotential (V)	−1 mA cm^−2^	*η*_1_	2.55 × 10^−2^	−2.71 × 10^−1^	0.279
	−3 mA cm^−2^	*η*_3_	1.61 × 10^−2^	−3.04 × 10^−1^	0.152
	−10 mA cm^−2^	*η*_10_	9.60 × 10^−3^	−3.64 × 10^−1^	0.048
	−30 mA cm^−2^	*η*_30_	1.30 × 10^−3^	−4.39 × 10^−1^	0.001
	−50 mA cm^−2^	*η*_50_	−9.20 × 10^−3^	−4.78 × 10^−1^	0.033
Local Tafel slope (V dec^−1^)	−0.6 mA cm^−2^	*b*_0.6_	1.06 × 10^−1^	−5.12 × 10^−2^	0.505
	−1.0 mA cm^−2^	*b*_1_	3.68 × 10^−2^	3.71 × 10^−2^	0.552
	−2.0 mA cm^−2^	*b*_2_	1.57 × 10^−2^	7.71 × 10^−2^	0.649
	−3.0 mA cm^−2^	*b*_3_	1.67 × 10^−2^	8.59 × 10^−2^	0.682 *
	−5.0 mA cm^−2^	*b*_5_	1.33 × 10^−2^	1.08 × 10^−1^	0.369
	−10 mA cm^−2^	*b*_10_	1.31 × 10^−2^	1.35 × 10^−1^	0.245
Current density (mA cm^−2^)	−0.3 V	*j*_0.3_	−1.61	−2.36	0.184
	−0.4 V	*j*_0.4_	−1.71	−17.9	0.024
	−0.5 V	*j*_0.5_	4.10	−61.0	0.043

**Table 2:** Log-linear regression parameters for OER performance metrics as a function of grain size. The regression was performed using *Y* = *A* log(*d*/μm) + *B* for annealed specimens, excluding the as-HRDSRed specimen. Superscript # indicates the best overpotential descriptor.

Parameter	Fixed Condition	Symbol	Slope, *A*	Intercept, *B*	*R* ^2^
Overpotential (V)	3 mA cm^−2^	*η*_3_	1.11 × 10^−2^	2.84 × 10^−1^	0.557
	10 mA cm^−2^	*η*_10_	1.09 × 10^−2^	3.10 × 10^−1^	0.715 ^#^
	30 mA cm^−2^	*η*_30_	1.15 × 10^−2^	3.30 × 10^−1^	0.416
Local Tafel slope (V dec^−1^)	2.0 mA cm^−2^	*b*_2_	−5.06 × 10^−2^	−1.12	0.113
	3.0 mA cm^−2^	*b*_3_	−2.11 × 10^−2^	−1.24	0.072
	5.0 mA cm^−2^	*b*_5_	−5.43 × 10^−3^	−1.31	0.006
	10 mA cm^−2^	*b*_10_	5.41 × 10^−3^	−1.35	0.002
Current density (mA cm^−2^)	0.27 V	*j*_0.27_	−3.63 × 10^−1^	1.71	0.436
	0.30 V	*j*_0.3_	−1.41	5.23	0.638
	0.33 V	*j*_0.33_	−8.44	26.1	0.787

## Data Availability

The original contributions presented in this study are included in the article. Further inquiries can be directed to the corresponding author.
